# Application of Concave Microwells to Pancreatic Tumor Spheroids Enabling Anticancer Drug Evaluation in a Clinically Relevant Drug Resistance Model

**DOI:** 10.1371/journal.pone.0073345

**Published:** 2013-09-10

**Authors:** Sang-Eun Yeon, Da Yoon No, Sang-Hoon Lee, Suk Woo Nam, Il-Hoan Oh, Jaehwi Lee, Hyo-Jeong Kuh

**Affiliations:** 1 Lab of Onco-Pharmacology and Experimental Therapeutics, Department of Biomedical Sciences, College of Medicine, The Catholic University of Korea, Seoul, Republic of Korea; 2 Department of Pathology, College of Medicine, The Catholic University of Korea, Seoul, Republic of Korea; 3 Catholic High Performance Cell Therapy Center, College of Medicine, The Catholic University of Korea, Seoul, Republic of Korea; 4 Cancer Evolution Research Center, College of Medicine, The Catholic University of Korea, Seoul, Republic of Korea; 5 Department of Biomedical Engineering, College of Health Science, Korea University, Seoul, Republic of Korea; 6 College of Pharmacy, Chung-Ang University, Seoul, Republic of Korea; Northwestern University, United States of America

## Abstract

Intrinsic drug resistance of pancreatic ductal adenocarcinoma (PDAC) warrants studies using models that are more clinically relevant for identifying novel resistance mechanisms as well as for drug development. Tumor spheroids (TS) mimic in vivo tumor conditions associated with multicellular resistance and represent a promising model for efficient drug screening, however, pancreatic cancer cells often fail to form spheroids using conventional methods such as liquid overlay. This study describes the induction of TS of human pancreatic cancer cells (Panc-1, Aspc-1, Capan-2) in concave polydimethylsiloxane (PDMS) microwell plates and evaluation of their usefulness as an anticancer efficacy test model. All three cell lines showed TS formation with varying degree of necrosis inside TS. Among these, Panc-1 spheroid with spherical morphology, a rather rough surface, and unique adhesion structures were successfully produced with no notable necrosis in concave microwell plates. Panc-1 TS contained growth factors or enzymes such as TGF-β1, CTGF, and MT1-MMP, and extracellular matrix proteins such as collagen type I, fibronectin, and laminin. Panc-1 cells grown as TS showed changes in stem cell populations and in expression levels of miRNAs that may play roles in chemoresistance. Visualization of drug penetration and detection of viability indicators, such as Ki-67 and MitoSOX, were optimized for TS for quantitative analysis. Water-soluble tetrazolium (MTS) and acid phosphatase (APH) assays were also successfully optimized. Overall, we demonstrated that concave PDMS microwell plates are a novel platform for preparation of TS of weakly aggregating cells and that Panc-1 spheroids may represent a novel three-dimensional model for anti-pancreatic cancer drug screening.

## Introduction

Pancreatic ductal adenocarcinoma (PDAC) is one of the most lethal types of cancer. Despite improvement in diagnosis and treatment, most patients are not candidates for curative surgical resection and the prognosis remains poor [1]. Only a minority (25∼30%) of patients respond to standard gemcitabine (GEM)-based treatments [2]. Despite much effort to find more effective therapeutic agents, novel agents or regimens have not yet been developed for PDAC. A characteristic feature of PDAC is intrinsic resistance to chemotherapy, which is mediated by various factors such as hypovascularization, prominent desmoplasia, expression of drug metabolizing enzymes, and recently suggested the presence of putative pancreatic cancer stem cells [3], [4]. Lack of appropriate models to produce clinically relevant efficacy data has been an important issue in pancreatic cancer therapeutics research.

The commonly used *in vitro* testing methods for anticancer drug efficacy typically involve growing cancer cell lines in monolayers on culture plastics [5]. Monolayer culture has remained a poor predictor of whether a given drug will ultimately yield clinical benefit due to the remote resemblance of monolayer cultures to the *in vivo* condition. Common animal models employed in drug testing for solid tumors are subcutaneous human tumor xenografts in nude mice [6]. However, the use of animal models in drug development studies presents disadvantages with feasibility as well as ethical concerns due to discomfort and pain caused to live subjects. Many researchers have therefore sought to address many of the problems associated with monolayer cultures as well as with animal models by creating three dimensional (3D) *in vitro* tumor models that better mimic *in vivo* tumor biology.

Three-dimensional *in vitro* tumor models of human solid tumors mimic *in vivo* tumor conditions known to contribute to multicellular resistance of human solid tumors, including 3D architecture, abundance of extracellular matrix (ECM), and cell–cell and cell–ECM communication Unlike monolayer systems, 3D *in vitro* tumor models have been successfully used to evaluate efficacy and tissue pharmacokinetics (PK) of anticancer drugs [7]–[9]. Since the chemoresistance of PDAC has been attributed to microenvironmental factors, 3D *in vitro* tumor models represent a promising approach for novel drug screening against PDAC. Tumor spheroids (TS, multicellular spheroid) are one of the most common 3D *in vitro* tumor model used to study the PK and pharmacodynamics (PD) of anticancer drugs [10]–[12].

A variety of methods have been employed to grow 3D spheroids [13]. Essentially, the available methods exploit conditions where adhesive forces between cells are greater than the adhesive attraction to the substrate on which the cells are plated. In the simplest form, this may involve a liquid overlay method, such as plastic tissue culture where the surface is coated with a thin layer of agarose or other substrates that will prevent the deposition of a matrix and cell attachment [14]. TS has been made by various other methods including spinner flask methods, gyratory rotation systems, hanging drop cultures, surface-modified substrates or scaffolds, and micro-fabricated microstructures. Each method has advantages and limitations [13], [15]. Perhaps the most widely used method to culture TS is the liquid overlay method in a 96 well plate coated with a non-adherent surface; this method has been used successfully with many cell lines. However, culture of some cell lines with weak aggregation properties was less successful with this conventional method. Pancreatic cancer cell lines such as Panc-1, Aspc-1, Capan-1, Capan-2 and Miapaca-2 showed the same issue as did non-aggregating cells, *i.e.*, TS formation by these cells was not successful in our preliminary experiments using agarose-coated 96 well U-bottom plates. Hence, novel methods using microstructures or artificial matrixes have been considered [16], [17]. Recently, microtechnology using biocompatible polymers, such as polyethylene glycol (PEG) and polydimethylsiloxane (PDMS), have created an opportunity to create micro-multiwell plates that are surrounded by adhesion-resistant surfaces [8], [18]. These microwell formats may provide better environments for TS formation, especially for cells with weak aggregation forces.

**Figure 1:**
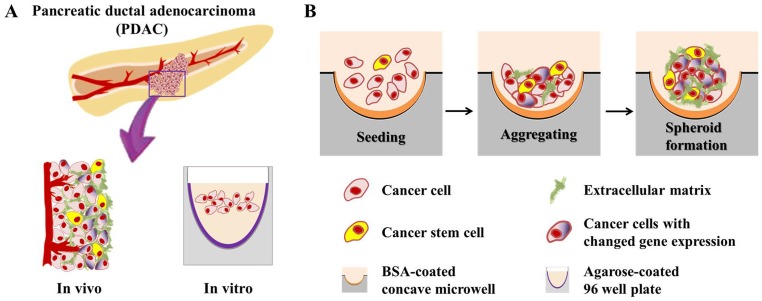
Schematic overview of pancreatic tumor spheroids (TS) generated in concave microwells. Pancreatic cancer cells cultured in agarose-coated 96 well plates produced only loose aggregates (A). The TS formation process in a BSA-coated concave microwell plate (B). The TS structure, compared with an *in vivo* tumor, showed a close resemblance to the *in vivo* condition. Cells in the TS retained the characteristics of *in vivo* tumors under 3D culture conditions.

In the present study, concave PDMS microwells previously developed for culture of hepatospheres and embryoid bodies [19], [20] were employed as a TS culture platform for weakly aggregating pancreatic cancer cells (Fig 1). The Panc-1 human pancreatic cell line showed stable sphere formation in these concave microwells within 5 days of culture, but culture was unsuccessful in 96 well plates. We evaluated the 3D construct of Panc-1 for resemblance to *in vivo* cellular morphology, gene expression, and microenvironmental characteristics. The spheroids in the concave microwells formed a fully compact structure sufficient for evaluation of drug penetration and antiproliferative activity using appropriate assay methods. Overall, our study showed that the use of concave microwell platform for pancreatic TS provides advantages of uniform size distribution in a size-controllable manner and with a relatively short duration of culture. The concave surface topography enables the stable formation of a single spheroid per well and allows easy harvest. Therefore, application of concave microwells for pancreatic TS will be useful for the development of high throughput screening platforms to identify novel therapeutic agents, key mechanisms of drug resistance, or therapeutic targets against human pancreatic cancer in more clinically relevant 3D models *in vitro*.

## Materials and Methods

### Cell lines and culture

The human pancreatic cancer cell lines Panc-1, Aspc-1, Capan-2 and Miapaca-2, and the human colorectal cancer cell line HT-29 were obtained from ATCC. Panc-1 and Miapaca-2 cells were grown in Dulbecco's modified Eagle medium with high glucose and Aspc-1, Capan-2 and HT-29 cells in RPMI-1640 medium (Gibco, Grand Island, NY) supplemented with 10% heat-inactivated fetal bovine serum (Welgene, Seoul, Korea).

### Chemical reagents

Doxorubicin (DOX) and GEM were kindly provided by Ildong Pharmaceutical Co., Ltd. (Seoul, Korea) and Boryung Pharmaceutical Co., Ltd. (Seoul, Korea), respectively. CellTiter 96® Aqueous MTS reagent powder was purchased from Promega Co. Ltd, (Madison, WI). Phenazine methosulfate (PMS), sodium acetate buffer solution (3 M), Triton-X-100 and other reagents were purchased from Sigma Aldrich Co. (St. Louis, MO), unless otherwise specified.

### Fabrication of concave PDMS microwell plates and pancreatic TS formation

Spheroids were generated using concave microwell plates fabricated using thin PDMS membranes. The concave microwell plates were prepared using a simple 3D microfabrication technique, as previously described [18]. Briefly, negative pressure was applied to an acrylic chamber to deflect the SU-8 prepolymer on the PDMS membrane to form convex SU-8 structures. The concave PDMS microwell structure was fabricated by replication of the convex SU-8 mold structure. In this study, we used three kinds of concave microwell plates (9 mm×9 mm×4 mm, W×D×H) having different numbers and diameters of concave wells: 64 wells/350 μm, 100 wells/600 μm, and 42 wells/900 μm. We previously reported that concave curvatures of these sizes enhanced the self-aggregation of diverse cells including hepatocytes, neurons, embryonic stem cells, and pancreatic cells [19], [21]–[23]. All microwell plates were pre-coated with 3% (w/v) bovine serum albumin (BSA) to minimize cell attachment before use. BSA-coated microwell plates were seeded with 1×10^5^ cells/200 μL per plate using a gentle loading technique so that cells precipitated into and were trapped within the microwells. After 10 min, a gentle flow of culture media was applied to remove cells remaining outside the wells. Specimens for scanning and transmission electron microscopic observation (SEM, TEM) were prepared as described previously [24].

### MitoSOX superoxide indicator for viability assay

MitoSOX^TM^ Red superoxide indicator (MitoSOX, Molecular Probes, Eugene, OR) was used for the measurement of viability changes of the TS, according to manufacturer's instructions. After drug exposure, spheroids were incubated with 5 μM MitoSOX for 30 min at 37°C, protected from light. Spheroids were then harvested on a coverslip and observed with a confocal microscope (LSM 510 Meta, Zeiss, Germany).

### MTS and acid phosphatase (APH) assay

TS cultured for 5 days in concave microwell plates were transferred to 96 well plates. For monolayers, 5×10^3^ cells per well were seeded. The MTS assay was run by adding 20 μL of MTS/PMS solution at the end of drug exposure, incubating the plates at 37°C for 4 h, and then reading the absorbance at 490 nm. The viable fraction was defined as ratio of absorbance of the drug-treated group to that of a control group receiving no treatment. For APH assay [25], the TS or cell pellet was carefully washed with PBS after drug treatment and incubated with 100 μL of assay buffer (0.1 M sodium acetate, 0.1% Triton-X-100 in deionized water and supplemented with p-nitrophenyl phosphate (Pierce, Rockford, IL)) at 37°C for 90 min. Following incubation, 10 μL of 1 N NaOH was added to each well, and absorption at 405 nm was measured on an ELISA reader within 15 min.

### Immunohistochemical staining

Immunohistochemical staining for TGF-β1, CTGF, MT1-MMP, collagen type I, fibronectin, and laminin was carried out using the respective primary antibodies and Dako EnVision™ Detection System (K5007). Paraffin-embedded spheroids were cut into 3 μm thick sections, deparaffinized and rehydrated. For antigen retrieval, a pressure cooker in microwave method was performed with a Target Retrieval Solution pH 9.0 (S2375). After gentle washing, nonspecific binding was blocked using 10% normal goat serum for 60 min. Sections were incubated with the primary antibodies against TGF-β1 (1∶200, Abcam, Boston, MA), CTGF (1∶400, Abcam), MT1-MMP(1∶40, Abcam), collagen type I (1∶200, Abcam), fibronectin (1∶250, Abcam) and laminin (1∶100, Sigma-Aldrich) at 4°C in a humidified chamber overnight. After blocking of endogenous peroxidase activity and washing in tap water or TBS, slides were counterstained with hematoxylin and mounted. Final images were obtained using a microscope (AX70, Olympus, Japan).

### DOX distribution and immunofluorescence staining for Ki-67 expression

DOX distribution in the TS was confirmed by slicing the spheroids into 10 μm thick frozen sections using O.C.T compound. DOX autofluorescence was detected using an inverted microscope (Axiovert 200 M, Carl Zeiss, Germany). For detection of Ki-67 expression, paraffin-embedded DOX-treated spheroids were cut into 3 μm thick sections and stained with primary antibody against Ki-67 (1∶50, Santa Cruz, Dallas, TX) and using the Dako EnVision™ Detection System. Briefly, sections were incubated with primary antibody at 4°C in a humidified chamber, then exposed to goat anti-rabbit IgG tagged Alexa488 fluorescein (1∶200, Molecular Probes) for 90 min at room temperature. After mounting the samples, fluorescence images were obtained using an inverted microscope equipped with a camera and processed using an Axio Vision Rel. 4.8 software system.

### Flow cytometry

Pancreatic cancer stem cell marker analysis was performed on single cell suspensions. The TS were first disaggregated by incubating them with 0.025% trypsin-EDTA solution at 37°C for 5 min. Simultaneous triple staining was done to determine a triple positive cell population. The antibody mixture of PE anti-human CD44 (BD Bioscience, San Jose, CA), FITC anti-human CD24 (BD Bioscience) and APC anti-human epithelial specific antigen (ESA, BD Bioscience) was added and incubated on ice in the dark for 20 min and stained cells were subjected to flow cytometry (BD FACSCanto^TM^ II equipped with BD FACS Diva software, Franklin Lakes, NJ). Respective isotype control antibodies were used at the same concentrations according to the manufacturer's instructions. Side and forward-scatter profiles were used to eliminate cell doublets.

### MicroRNA expression profiling

Total RNA was isolated from monolayers or TS using Trizol (Invitrogen, Carlsbad, CA). Single cell preparation from TS was carried out as described under “Flow cytometry.” RNA quality control was performed by using the Experion^TM^ system (Bio-Rad, Hercules, CA). MicroRNA (miRNA) expression profiling analysis was performed using a human miRNA microarray (manufactured in Functional RNomic Research Center, The Catholic University of Korea) containing 1,585 miRNA probes selected from the public database, miRBase v16 (GenoExplorer Microarray Platform, GenoSensor Corp., Tempe, AZ). Hybridization and scanning of the microarrays were performed according to standard protocols. Large-scale miRNA expression profiling was performed as described previously [26]. The microarray data were analyzed for DEG findings and hierarchical cluster criteria using a GenPlex V3.0 (Istech Inc., Gyeong-gi, Korea). After normalization and class-specific filtering, total expression of 496 miRNAs were subjected to unsupervised clustering analysis and the Pearson's correlation coefficient was calculated. TreeView programs (Stanford University) were utilized for visualization.

### Quantitative RT-PCR (qRT-PCR)

Total RNA extracted from the same samples used for miRNA microarrays was used for qRT-PCR, which was carried out with Mir-X miRNA First-strand synthesis and SYBR Green Real time PCR Master Mix (Clontech Laboratories, Inc., Mountain View, CA) according to the manufacturer's instructions. The housekeeping gene U6 was used for standardization of the initial miRNA content of each sample. Relative changes in gene expression were expressed as fold changes calculated by the following formula: fold change  = 2^−ΔΔCt^, where ΔΔCt  =  (Ct of gene of interest, 3D – Ct of housekeeping gene, 3D) – (Ct of gene of interest, 2D – Ct of housekeeping gene, 2D), and Ct is the threshold cycle number.

### Statistical analysis

Statistical analyses were carried out using Microsoft Excel 2010. ANOVA with Tukey post hoc or t-test was used to test the statistical significance, where p<0.05 were considered statistically significant.

## Results

### TS formation using concave microwell plates

**Figure 2:**
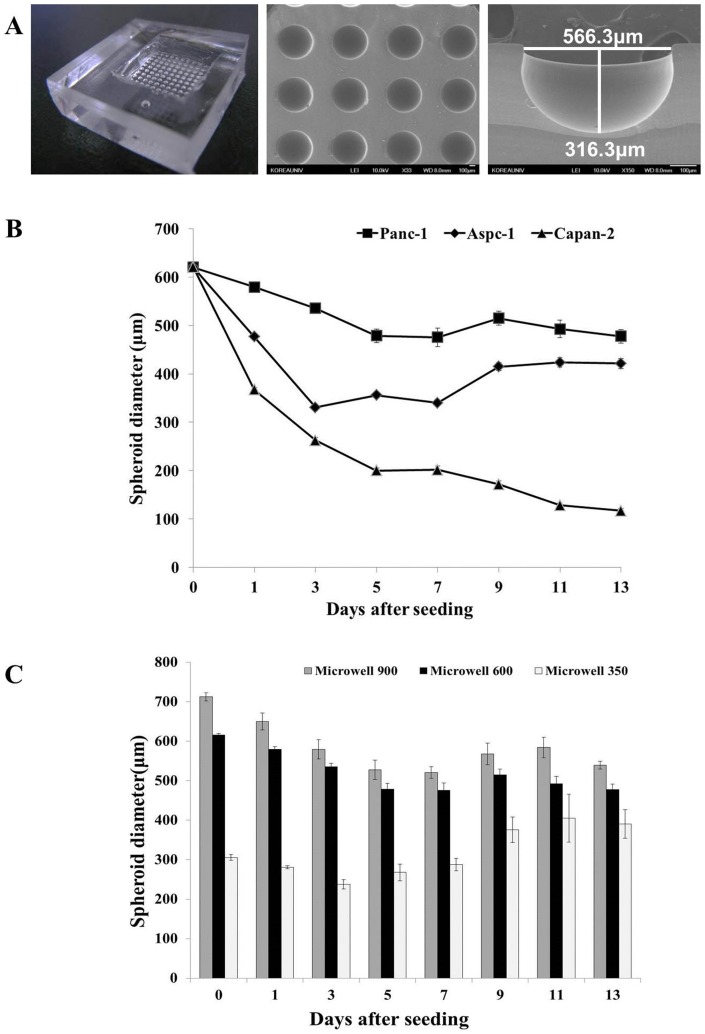
Concave PDMS microwell plate for culture and growth of pancreatic tumor spheroids (TS). Shape and dimension of concave microwell plate 600 (A). Changes in size of three different pancreatic spheroids cultured in microwell 600 (B). Size distribution of Panc-1 spheroids cultured in three different sized-microwell plate during 13 days of culture (C). Data are expressed as mean ± SE of a minimum of 10 spheroids cultured in one microwell plate.

Pancreatic TS was generated by preparing and using concave PDMS microwell plates as described in the Materials and Methods. SEM images of concave microwell plate 600 showed 100 microwell arrays with diameters of 566.3 μm and depths of 316.3 μm (Fig. 2A). Two other types of microwell plates (900 and 350) contained 42 and 64 microwell arrays with well dimensions of 860 μm and 330 μm in diameter and 430 μm and 150 μm in depth, respectively.

We compared the spheroid formation and growth of four different human pancreatic cancer cell lines (Panc-1, Aspc-1, Capan-2, Miapaca-2) in microwell plate 600 over 13 days (Fig. 2B). Panc-1 and Aspc-1 spheroids showed similar growth profiles, *i.e.*, the TS size decreased until 5∼7 days of culture, then increased afterword. The initial reduction in size was significantly greater in Aspc-1 than Panc-1 TS, hence, the size of Aspc-1 spheroids reached 360 μm (75% of Panc-1 TS) at 13 days. Capan-2 spheroids showed a continuous decrease in size and eventually reached 110 μm which was only 25% of Panc-1 TS. Miapaca-2 cells didn't form spheroids and loose aggregates formed were not stable enough for harvest and further experiments.

We determined the effects of concave microwell dimensions on Panc-1 TS formation by comparing the sizes of TS cultured in microwell plates over 13 days. All three types of concave microwell plates showed good formation efficacy, as high as 90∼100%. Cells seeded in microwell plates 600 and 900 showed a decrease in size of the cell aggregates from 600 μm and 715 μm at day 0 to 480 μm and 520 μm at day 5, respectively, or an approximately 24% size reduction (Fig. 2C). The sizes of the spheroids were subsequently maintained until the end of culture, with small fluctuations. On the other hand, the size of aggregates formed in microwell plate 350 was 300 μm in diameter and the size was maintained for 7 days. The size of the spheroids then reached 400±35 μm at 13 days of culture, or a 133% increase (p<0.05). We selected Panc-1 cells with microwell plate 600 (566 μm ×316 μm) for further experiments, which gave an optimal size of spheroids with small variation (480±10 μm at day 5).

**Figure 3:**
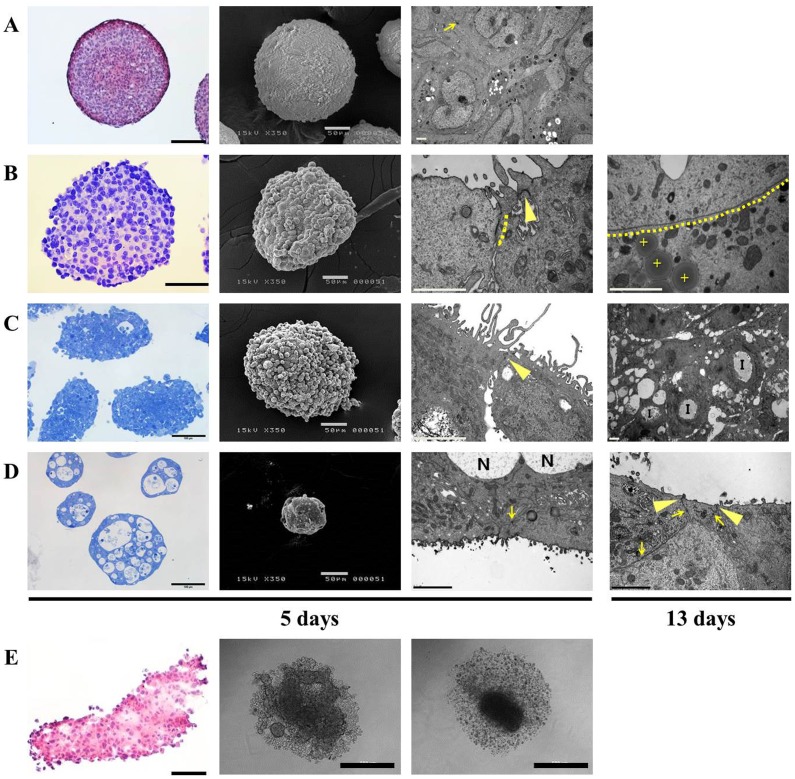
Morphology and histological examination of tumor spheroids (TS) cultured in concave microwell 600 or in 96 well plates. Representative images of H&E stained paraffin sections or toluidine blue stained semi-thin sections, SEM and TEM images of HT-29 (A), Panc-1 (B), Aspc-1 (C), and Capan-2 (D) spheroids cultured in concave microwell 600 plates for 5 and 13 days. Aggregates of Panc-1, Aspc-1, and Capan-2 cells formed in agarose-coated 96 well plates shown as an H&E stained paraffin section or bright field images (E). Arrow: desmosome; dotted lines: gap junctions; arrow head: tight junction; cross: lipid droplet; I: invagination structure; N: necrotic regions. The scale bars indicate 100 μm, 50 μm, 2 μm and 500 μm, in H&E or toluidine blue stained, SEM, TEM and bright-field images, respectively.

The morphology of pancreatic spheroids cultured in concave microwell plate 600 were compared with that of HT-29 spheroids. HT-29 cells are commonly used for 3D cultures such as TS and multicellular layers (MCL) in many studies [10], [12], [24]. HT-29 cells produced spheroids 300 μm in diameter after 5 days of culture in microwell plate 600. The HT-29 spheroids showed a rather smooth surface, with compact organization of the interior cells (Fig. 3A). Panc-1 spheroids were also spherical aggregates with compact structure but the surface was quite rough, with bulging presentation of cells on the peripheral layers, and this morphology was maintained even after 13 days of culture (Fig. 3B and S1A). Five-day-cultured spheroids of Panc-1 were 350∼400 μm in size, with approximately 22 cell layers. After 13 days of culture, the spheroids still showed similar sizes of 380∼430 μm diameter and 24 cell layers, indicating insignificant growth after 5 days (Fig. S1A). Note that sizes measured during culture in microwells were different from those of fixed samples. Cells grown in 3D condition with direct cell-cell and cell-ECM interaction usually develop typical adhesion structures of cell membranes. Consequently, tight junctions and gap junctions were observed in the peripheral cell layers of the Panc-1 spheroid aggregates. In particular, gap junctions were prominent in Panc-1 spheroids by 13 days of culture and these appeared over a large region (Fig. 3B). By 13 days, Panc-1 spheroids also showed a disorganized structure of the intracellular organelles with lipid vesicles, suggesting the induction of necrotic or apoptotic processes. Aspc-1 spheroids cultured for 5 days showed a rough surface as Panc-1 spheroids (Fig. 3C). Tight junctions and unique invagination structures with microvilli were observed (Fig. S1B) and many vacuoles were seen in the cytosol. In the same culture condition, Capan-2 spheroids showed very unique morphology; the TS were full of necrotic regions and tight junctions and desmosome were fully developed (Fig. 3D and S1C). The drastic size reduction of Capan-2 TS (Fig. 2B) may be associated with these necrotic regions induced during 3D culture.

Panc-1, Aspc-1, and Capan-2 cells failed to form TS in agarose-coated 96 well plates, instead, formed loose aggregates, which may be attributed to weak cell-cell aggregation forces (Fig. 3E). Among these three cell lines, we confirmed that stable formation of Panc-1 spheroids can be achieved in 5 days with the concave microwell plate 600. These spheroids showed appropriate sizes (average of 400 μm) and formation efficiencies (over 90%) for use as drug screening 3D models. The concave PDMS microwells plates are therefore a very useful platform for spheroids formation even for weakly aggregating cells such as Panc-1cells.

### Expression of growth signaling proteins and ECM components

**Figure 4:**
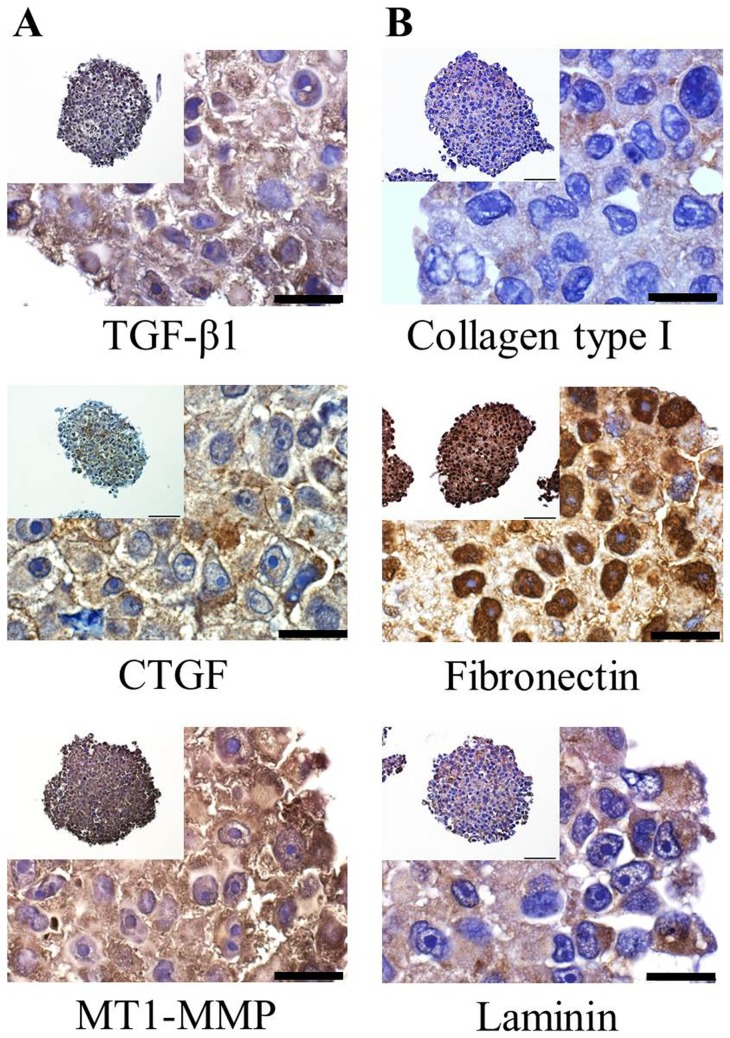
Expression of proteins associated with tumor growth and drug resistance. Spheroids were cultured for 5-β1, CTGF, and MT1-MMP (A) and for ECM proteins such as collagen type I, fibronectin, and laminin (B). (Scale bar  = 20 μm).

Tumor cells secrete a variety of proteins including growth factors and ECM proteins; these sustain proliferation potential as well as confer drug resistance. Realistic mimicry of the *in vivo* condition of human tumors therefore requires the expression of these proteins. The immunohistochemically stained sections of Panc-1 spheroids showed expression of TGF-β1, CTGF, MT1-MMP, collagen type I, fibronectin and laminin (Fig. 4A, B). TGF-β1, CTGF, and MT1-MMP were detected in the cytoplasmic as well as extracellular spaces. These expressions were seen throughout the spheroids, but expression of TGF- β1 was most pronounced in the outer layers.

Collagen type I, fibronectin and laminin are the major components of ECM in tumor tissue and Panc-1 spheroids showed expression of these ECM proteins. The ECM proteins expressed in the extracellular space were short fibrous forms for all three of these proteins. In particular, strong nuclear staining was noted for fibronectin throughout the whole area of the spheroids. Taken together, these results confirmed the *in vivo* tumor-like condition of 5-day-cultured Panc-1 spheroids based on the expression of growth signaling molecules and ECM proteins. Accordingly, the use of concave PDMS microwells for culture of Panc-1 spheroids appears to be a novel platform for spheroid formation that can be used as a 3D drug screening model for pancreatic cancers.

### The pancreatic cancer stem cell population in 3D cultures

**Figure 5:**
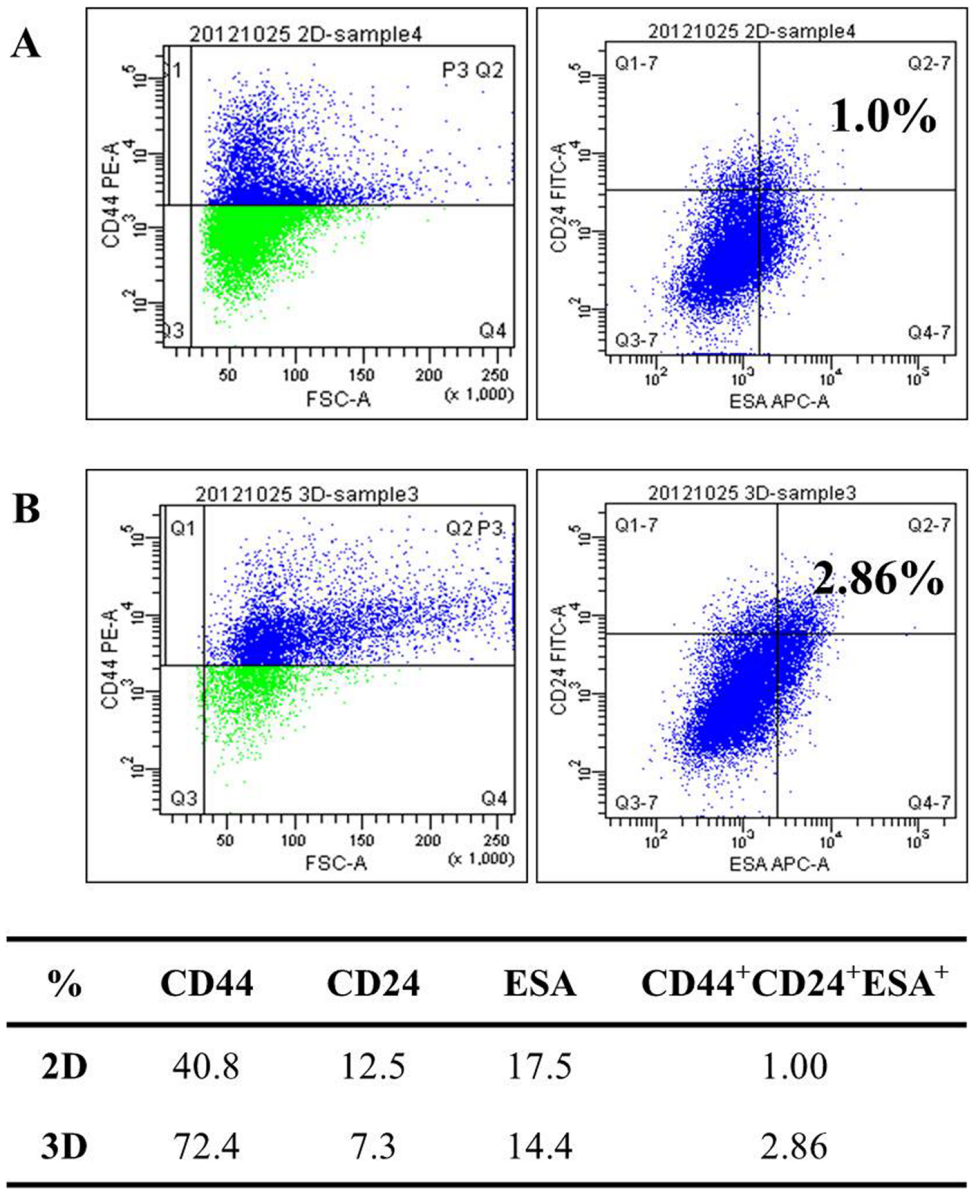
Analysis of pancreatic cancer stem cell markers. Expression patterns of stem cell markers such as CD44, CD24, and ESA were compared between 2D (A) and 3D cultures (B). Percentages of the cell population expressing CD44, CD24, and ESA in Panc-1 cells cultured under 2D and 3D conditions are summarized in the table.

The spheroid/colony forming capacity is the most widely used method to identify stemness in cancer cell cultures. Recent studies reported that the number of cancer stem cells, determined by marker expression, changed after 3D culture of human cancer cell lines [27], [28]. The stem cell population in Panc-1 cells grown as 2D or 3D cultures is shown in Fig. 5. In the monolayers, 40.8% of the cells expressing the cell surface marker CD44, 12.5% expressed CD24, and 17.5% expressed ESA. When cells formed spheroids, this population profile changed, *i.e.*, CD44 expression increased to 72.4% while CD24 and ESA expression decreased to 7.3% and 14.4%, respectively. The cancer stem cells with triple positive makers, (CD44^+^CD24^+^ESA^+^ cells) comprised only 1.0% of the cells in monolayers, but this value showed a statistically significant increase to 2.9% in spheroids. These data indicated that cancer stem cell populations increased under 3D culture conditions, which may be one explanation for higher drug resistance in 3D cultures.

### MicroRNA expression profiling

**Figure 6:**
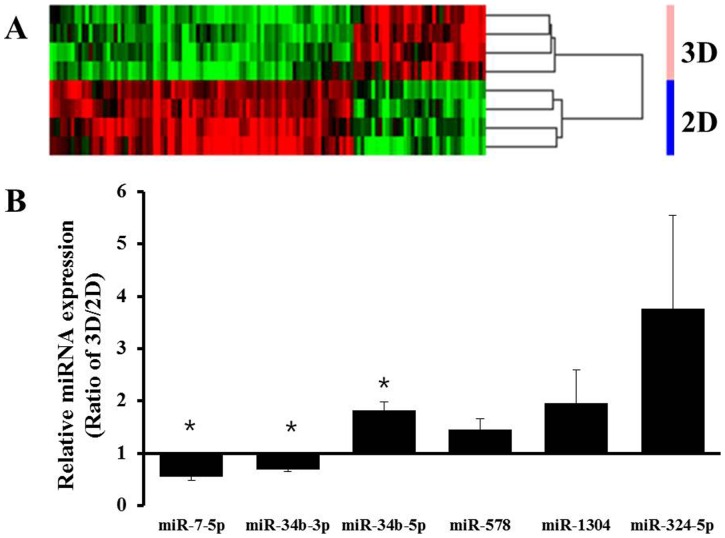
The miRNA expression profiles were compared between 2D and 3D cultures. A heatmap of differentially expressed miRNAs generated by unsupervised hierarchical cluster analysis (A). Differential expression levels of miRNAs between 2D and 3D cultures were confirmed using qRT-PCR for several genes selected from microarray data. Among the differentially expressed miRNAs, up-regulated miRNAs including miR-34b-5p, miR-578, miR-1304, and miR-324-5p and down-regulated miRNAs including miR-7-5p, and miR-34b-3p were analyzed using primers for mature mRNAs (B). Data are expressed as the mean ± SE of triplicates. * p<0.05, n = 3.

Recent evidence indicates that miRNAs regulate gene expression at the post transcriptional level by targeting mRNAs. Changes in expression levels of genes controlling drug metabolism or transport and drug target genes are strongly associated with acquisition of drug resistance; hence, the role of miRNAs as negative regulators of these genes has become a new focus of pharmacogenomics studies in cancer therapeutics [29]. Since gene expression changes have been reported in 3D cultures, miRNA expression changes are also of interest, as miRNA may play an important role in gene regulation associated with the chemoresistance associated with 3D cultures. Cluster analysis of our data obtained from gene chip assays for miRNA expression in Panc-1 cells suggested that many miRNAs were differentially expressed depending on the culture system used. The chip contained 1,585 human miRNAs and after the genes were filtered by present call, a total of 496 probes were available for further application in cross-correlation analysis. A number of miRNAs (as many as 121 miRNAs) showed differential expression. The clustering results of the overall expression patterns of miRNAs indicated down regulation of more genes in 3D than in 2D cultures (Fig. 6). Table 1 summarizes the miRNAs of Panc-1 cells that were up or down-regulated in spheroids compared to monolayers. A greater than 1.5 fold increase was observed for 36 miRNAs, including miR-136-5p and miR-34a-5p, and a lower than 1.5 fold decrease was seen for 85 miRNAs including miR-7-5p and miR-34b-3p in spheroids cultured on concave microwell plates. The differential expression of miRNAs from array experiments was confirmed by further analysis of selected miRNAs, including miR-7-5p, miR-34b-3p, miR-34b-5p, miR-578, miR-1304, and miR-324-5p, using qRT-PCR. miR-34b-5p showed 1.8 fold increases in expression, whereas miR-7-5p and miR-34b-3p showed 2.0 and 1.6 fold decreases in expression, respectively (p<0.05). Expression of miR-578, miR-1304, and miR-324-5p showed 1.5, 2.4, and 3.7 fold increase, respectively, but did not reach statistical significance. These findings were consistent with the miRNA array data. These data again indicate the significance of 3D cultures as tumor models for drug screening and chemoresistance studies, and suggest that genes with significant changes may be worth further investigation because of their possible involvement in the acquisition of drug resistance.

**Table 1:** Differentially expressed miRNAs in Panc-1 cell cultured under 2D and 3D conditions. Fold change indicates 3D intensity/2D intensity.

Gene	Fold change	P-Value
miR-1972	9.769	0.004
miR-566	5.049	<0.001
miR-1303	3.517	0.003
miR-136-5p	3.3	0.004
miR-1268	3.081	<0.001
miR-1299	2.102	0.006
miR-019a	2.063	0.049
miR-034a-5p	1.998	<0.001
miR-034b-5p	1.952	0.004
miR-578	1.822	0.019
miR-1304	1.715	0.002
miR-324-5p	1.58	0.004
miR-221	−1.834	0.007
miR-199a-5p	−2.965	0.001
miR-34b-3p	−2.977	0.002
miR-634	−3.063	0.003
miR-328	−3.22	<0.001
miR-212	−3.377	0.004
miR-34c-3p	−3.812	0.001
miR-7-5p	−3.958	0.006
miR-220a	−3.968	<0.001
miR-1229	−4.738	<0.001

### Drug penetration and distribution into spheroids

**Figure 7:**
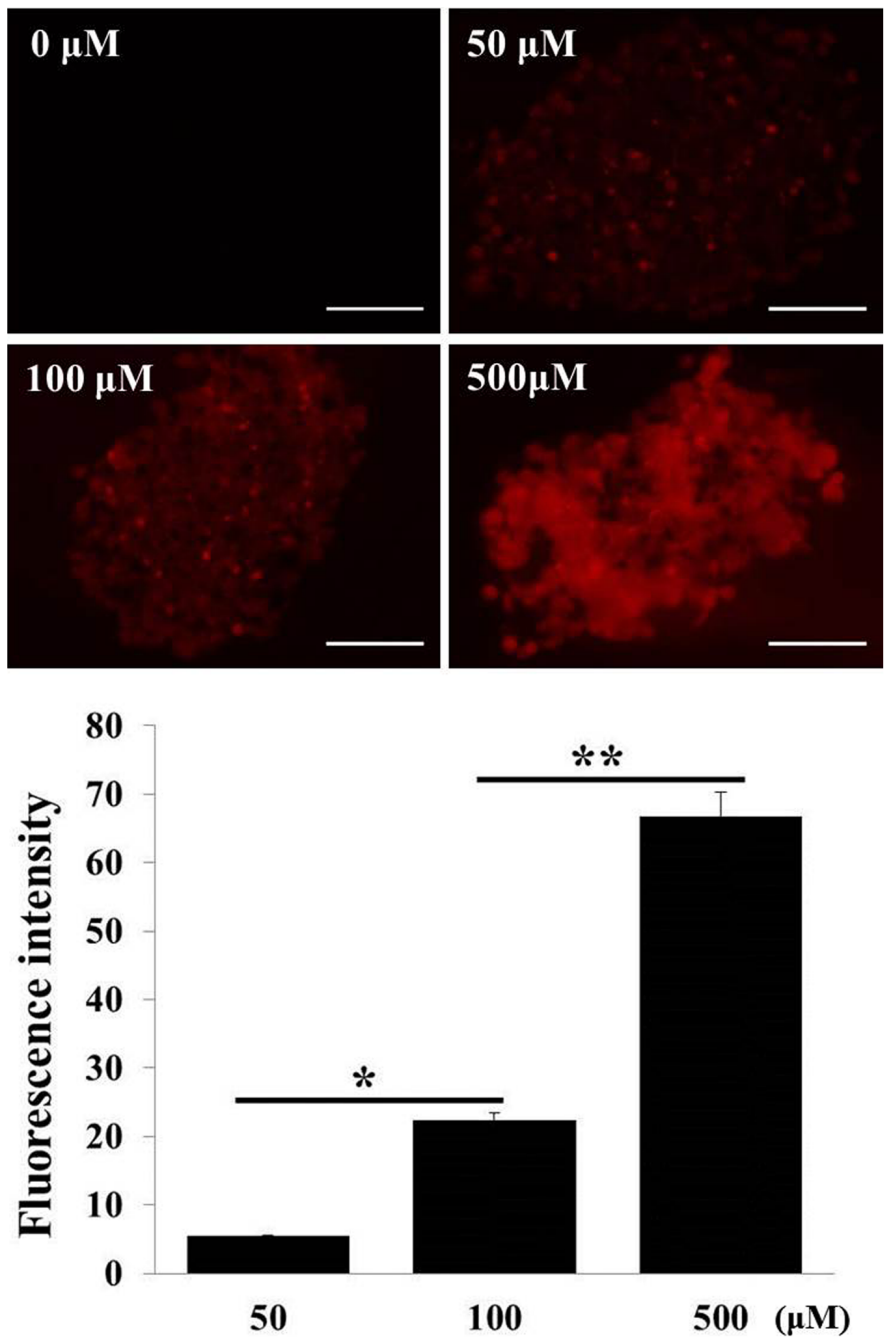
Penetration of DOX into Panc-1 spheroids. Penetration was evaluated by imaging DOX autofluorescence in sections of spheroids. Spheroids were cultured in a concave microwell 600 plate for 5 μM for 12 h. Fluorescence intensity across sections was measured and expressed as the mean ± SE of 5 replicates. (Scale bar  = 100 μm) * and **, p<0.01 and p<0.001, respectively.

Drug resistance arises due to decreased drug sensitivity in patient tumors, but drug delivery barriers and penetration kinetics are also important factors that contribute to the clinical efficacy of antitumor drugs. The 3D structure of an *in vivo* tumor with poor perfusion presents physical as well as physiological barriers for drug penetration, and can result in a low drug concentration around target cancer cells and therefore an incomplete response [30]. Spheroids are excellent models for the study of cellular accumulation and diffusion of drug in solid tumors and can be used to evaluate the PK-PD relationships of anticancer agents. We evaluated DOX penetration in Panc-1 TS using confocal fluorescence images (Fig. 7). DOX showed full penetration into the core of the spheroids within 12 h at all concentrations tested. Greater accumulation, as indicated by higher fluorescence intensity, was observed with higher dose exposure; however, the accumulation ratio, calculated by the ratio of intensity/dose, indicated a non-linear relationship between accumulation level and exposure level, which can be attributed to saturable uptake of DOX in Panc-1 cells.

### Cell viability assays for pancreatic TS

**Figure 8:**
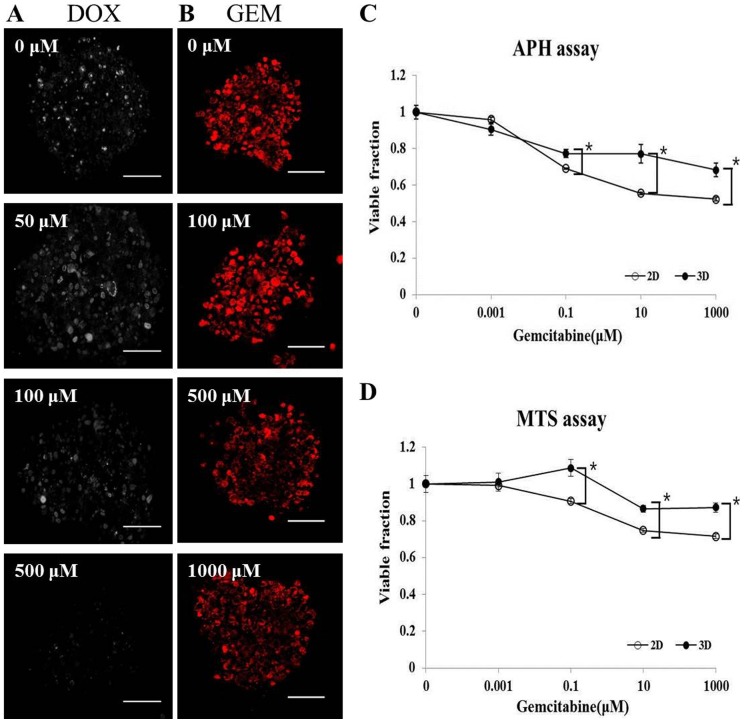
Cytotoxicity/viability assays for Panc-1 spheroids. Changes in Ki-67 expression were measured following exposure to DOX at the indicated concentrations for 12 h (A). Changes in MitoSOX were measured following exposure to GEM at the indicated concentration for 12 h (B). MTS (C) and APH assay (D) after exposure to GEM for 72 h. Data are expressed as the mean ± SE of 6 replicates. (Scale bar  = 100 μm) * p<0.001.

Cellular viability has been determined in spheroids using various methods. Viability assays for spheroids differ from classical methods for monolayer cultures due to factors related with the 3D structure. We used four different assays to test for viability changes in Panc-1 spheroids, including Ki-67 expression, MitoSOX staining, and MTS and APH assays (Fig. 8). After a 12 h treatment with 50, 100, or 500 μM of DOX, the number of proliferating Ki-67 positive cells decreased from 38% to 3% (Fig. 8A). The mitochondrial superoxide indicator MitoSOX also indicated a proportional decrease in viability after GEM treatment (Fig. 8B), but only a 30% decrease compared to control was observed at the highest concentration of 1 mM, suggesting GEM resistance in Panc-1 spheroids. These results, nonetheless, indicate that Ki-67 (+) cells or a MitoSOX signal can be a useful proliferative assay for spheroids. The MTS and APH assays were also feasible assay methods for spheroids (Fig. 8C, D). Both MTS and APH assays showed rather similar results of a shallow drug response relationship for GEM. After 72 h exposure to GEM, the IC_30_ values, based on the MTS assay was determined as 862 μM and >1 mM for monolayers and spheroids, respectively. The APH assay resulted in IC_30_ values of 0.614 μM and 178 μM for monolayer and spheroids, respectively, indicating GEM resistance of up to 30 fold. Comparison of the data derived from the MitoSOX, MTS, and APH tests indicated that all three methods showed consistent results suggestive of a fraction with quite high resistance (40∼60%). This highly resistant fraction was evident in both 2D and 3D cultures, but differential sensitivity was observed, indicating a contribution of cell-cell and cell-ECM interaction to drug resistance in cultures with 3D architecture. The degree of GEM resistance seemed to depend on the assay method utilized, but the 3D cultures of Panc-1 cells prepared in concave microwells showed overall drug resistance as expected and the two colorimetric assays are simple and appropriate for drug efficacy screening using spheroid cultures.

## Discussion

We showed that pancreatic spheroids were successfully cultured in concave microwell plates. Among three cell lines tested (Panc-1, Aspc-1, and Capan-2), Panc-1 formed most stable TS. The Panc-1 spheroids were structurally and functionally mature by day 5 and displayed greater resistance to GEM than monolayers did, suggesting that these spheroids and the concave microwell platform, along with assays for gene regulation and viability changes, could be useful for anticancer drug screening and studies of drug resistance. TS can be produced by a variety of methods, including the hanging drop method and liquid overlay methods using agarose or poly-hydroxyethyl methacrylate (poly-HEMA) [24], [31]. Recently, microwell plate fabrication techniques have incorporated photolithography to define microscale material patterns for use in the field of tumor biology [32].Among materials for microwell plate fabrication, PEG [16] and PDMS [19] have been shown to resist cell adhesion, allowing spheroid formation. While the processes underlying cell aggregation are similar to those occurring in hanging drops or liquid overlays, polymer-based microwell plate methods provide major advantages such as greater formation rate, less variation in size distribution, and the feasibility of automation with minimum cell loss [21]. The topography of the microwell surface significantly affects the stability, shape, and size distribution of the formed spheres, as shown by the hepatospheres and embryoid bodies. The advantages of concave over cylindrical wells have been demonstrated previously [19], [20]. The usefulness of concave microwells is extended to an *in vitro* 3D tumor model in the present study, where pancreatic TS have been successfully formed and their *in vivo*-like characteristics confirmed.

Application of the liquid overlay method in agarose-coated 96 well plates to human pancreatic cells resulted in a formation efficiency of less than 2%, which was in great contrast to other cell lines, such as HT-29 (data not shown). Our previous study showed that the microscale concave structure prevents cell adhesion, perhaps because concave microwells provide a greater chance for cells to collide with each other and aggregate toward spheroid formation [19]. In addition, increased concentrations of secreted autocrine growth factors may play key roles in spheroid formation. Therefore, the proposed concave PDMS microwell plates have unique advantages for the formation of TS that could enable the study of drug screening and resistance using weakly aggregating pancreatic cancer cells. Therefore, it is better than other currently available platforms.

When cells are cultured in a compact 3D architecture beyond the size of 500∼600 μm, cells may become quiescent and even undergo cell death inside spheroids due to a limited diffusion of oxygen, nutrients and other factors required for cell survival and proliferation [33]. Development of necrotic regions in TS depends not only on the size of spheroids but also on the cell lines as shown in the present study. In contrast to non-necrotic appearance of Panc-1 TS, other two cell lines (Aspc-1 and Capan-2) showed abundant necrotic regions resulting in the shrinkage of spheroid structure of Capan-2 TS (Fig. 3). These results suggest that a careful examination of spheroid structure is recommended for selection of TS model appropriate for anticancer drug screening and studies of drug resistance.

Panc-1 spheroids generated in concave microwells showed expression of drug resistance-related molecules, including TGF-β1, CTGF, MT1-MMP, collagen type I, fibronectin, and laminin (Fig. 4). TGF-β1, CTGF, and MT1-MMP are important molecules with well-known associations with poor prognosis and drug resistance in pancreatic cancer [34]. Tumor-associated stroma and ECM are also known to contribute to environment-mediated drug resistance (EMDR) [35]. The cell-adhesion mediated drug resistance (CAM-DR) phenomenon in particular is mediated by adhesion of tumor cell via integrins to stromal fibroblasts or to components of the extracellular matrix such as collagen, fibronectin, and laminin [35]. Based on the expression of these factors in spheroids cultured in concave microwell plates, microwell-grown TS are validated as appropriate models for the study of chemoresistance in pancreatic tumors.

Pancreatic cancer stem cells are commonly defined by expression of CD44, CD24, and ESA on the cell membrane surface [36]. The expression of these markers changes in response to different cell culture conditions, such as 2D versus 3D. Several studies have compared expressions of these markers but the results are contradictory. When Panc-1 cells were cultured as spheroids, the cell population expressing CD44 decreased and that expressing CD24 increased in one study [28], whereas another study showed that the CD44^+^CD24^+^ESA^+^ triple positive cell population increased (this finding is consistent with our data) [37]. As CD44 antigen have shown a correlation with chemoresistance [38], the presence of cancer stem cells expressing CD44 may contribute to the acquisition of growth potential as well as drug resistance. In this regard, changes in stem cell populations under 3D culture conditions may reflect the significance of spheroid culture as an important experimental model for drug resistance studies as well as for drug screening. At the same time, the point should be emphasized that novel platforms for spheroid culture will be in great demand in cancer research where our concave microwell plates may represent a prototype.

Recent studies have shown novel roles of miRNAs in the regulation of genes related with development, differentiation, proliferation, survival, and death [39]. Current evidence suggests that miRNAs may function as oncogenes or tumor suppressors, based on their target mRNAs, and that they can serve as biomarkers for prognosis and as pharmacogenomics markers for therapeutic responses [29]. We analyzed the miRNA expression profiles of Panc-1 cells and observed differential expression between cells growing as monolayers and as spheroids (Fig. 6). Among 121 miRNAs that showed significantly different expression levels, 36 miRNAs and 85 miRNAs were found to be up-regulated and down-regulated, respectively, upon spheroids formation. Changes in expression of miR-34 family [40] and miR-221 [41] have been reported to be associated with GEM resistance in pancreatic cancers. Our result also showed changes in these genes, *i.e.*, an increased level of miR-34b-5p and decreased levels of miR-34b-3p, miR-34c-3p, and miR-221 were observed in spheroids cultures of Panc-1 cells. These results emphasize the significance of 3D culture models for drug resistance studies and efficacy evaluation as well as the importance of our concave platform as a novel culture method for spheroids. The difference in the miRNA expression between stem vs. non-stem cell population in 2D and 3D cultures may be of interest with respect to their potential contribution to drug resistance. Although we have not done such analyses due to low % of stem cell population in Panc-1 cultures, *e.g.*, only 1% in 2D and 2.9% in 3D cultures, respectively, this may be worth studying further.

The PK-PD studies using 3D spheroids remain limited by the selection of antiproliferation assays to obtain dose-response relationships. Antiproliferative activity of drugs in 3D cultures can be evaluated by Ki-67 expression and also by using assays such as MTS, Cell Counting kit-8 (CCK-8), APH [25], MitoSOX, and calcein acetoxymethylester (Calcein AM) [10]. Ki-67 is a nuclear protein with activity that is associated with cell proliferation. Although widely used, its non-nuclear localization and its relationship with cell cycle phases make its use inappropriate in 3D *in vitro* cultures [42]. Calcein AM and MitoSOX are cell permeant dyes that are widely used for determination of cell viability, invasion, adhesion, and migration, but these require confocal or fluorescence microscopy, which may be time-consuming for quantitative assays. Therefore, we recommend the APH assay as an appropriate method for viability assays of cells in spheroids.

## Conclusions

Compared to the conventional monolayers or suspension cultures, 3D culture models have drawn attention as *in vivo* mimic models that can produce clinically relevant data; this is critical for successful development of new chemotherapeutics and potential targets. Among the several types of 3D culture models, TS is an appropriate model for study of penetration and efficacy of anticancer agents and several methods have been utilized for TS preparation from various cancer cells. Human pancreatic cancer cells, however, are weakly self-aggregating and do not readily form TS successfully with conventional methods. In this study, we demonstrated the potential use of concave microwell plates fabricated using PDMS for TS formation using three different human pancreatic cancer cell lines, Panc-1, Aspc-1, and Capan-2, and examined the *in vivo* mimicking characteristics of the TS formed by Panc-1 cells. Panc-1 TS with unique cell adhesion structures were successfully produced within 5 days of culture using concave microwells. Expression of growth factors and ECM proteins and changes in stem cell population and miRNA profiles, all of which are associated with mechanisms of drug resistance, were confirmed in cells grown as TS. Methods for quantitative analysis of drug penetration through cell layers and viability changes after drug treatment were successfully optimized for TS formed in microwell plates. Overall, we demonstrated that concave microwell plates are a novel platform for preparation of TS of poorly aggregating cells. This culture method could be applied to other fields of study where *in vitro* 3D tumor models are required.

## Supporting Information

Figure S1 **Morphology and histological examination of pancreatic tumor spheroids (TS) cultured for 13 days in concave microwell 600.** Representative images of H&E stained paraffin sections or toluidine blue stained semi-thin sections, SEM and TEM images of Panc-1 (A), Aspc-1 (B) and Capan-2 (C) spheroids. Cross: lipid droplets; N: necrotic regions; I: invagination structure. The scale bars indicate 100 μm, 50 μm, 2 μm and 500 μm, in H&E or toluidine blue stained, SEM, and TEM images, respectively.
(TIF)
