# Supplementary figures and images for: Application of Concave Microwells to Pancreatic Tumor Spheroids Enabling Anticancer Drug Evaluation in a Clinically Relevant Drug Resistance Model
Source: PLoS One. 2013 Sep 10;8(9):e73345. doi: 10.1371/journal.pone.0073345 (PMC3769301; doi:10.1371/journal.pone.0073345)

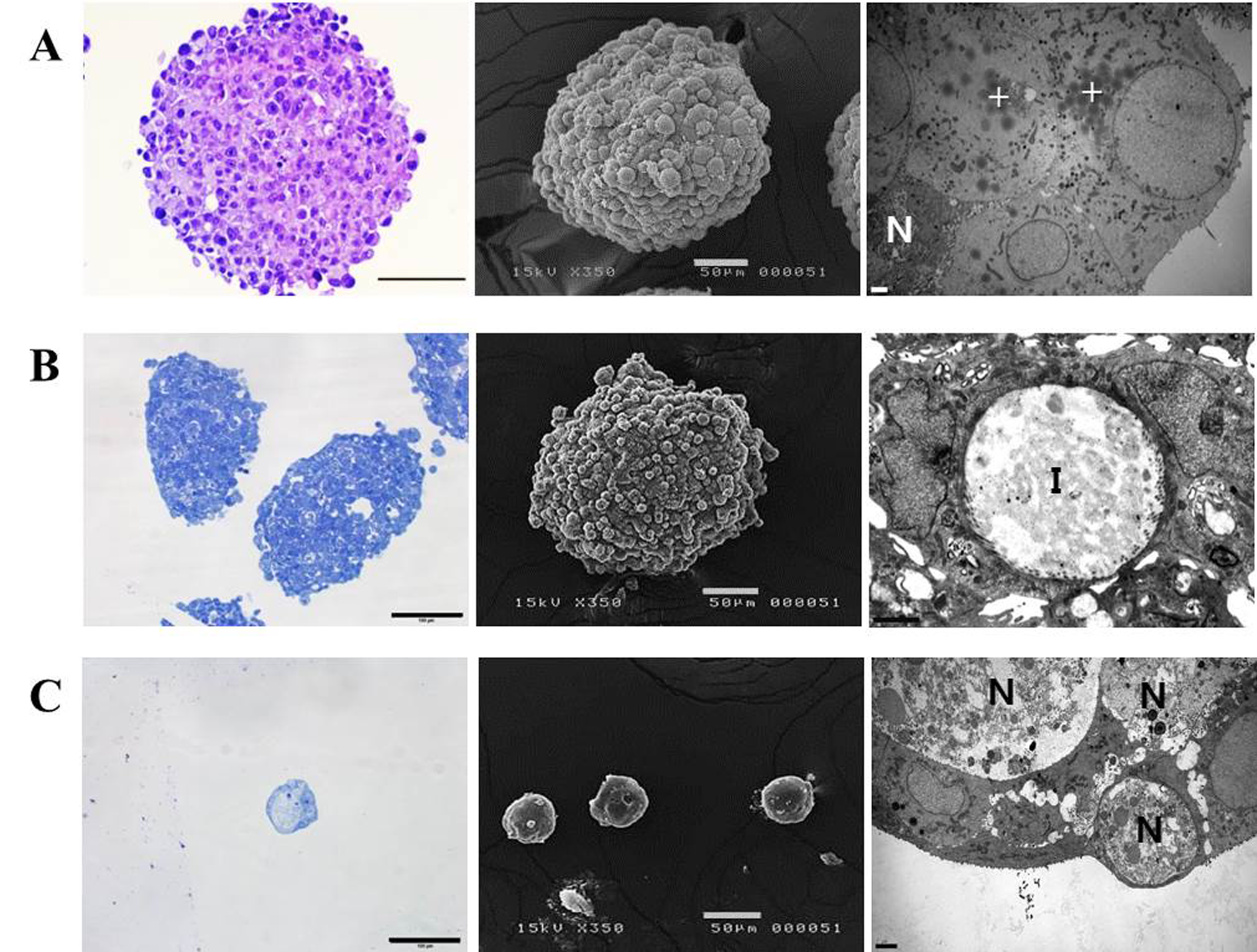

Supplement: Figure S1 — Morphology and histological examination of pancreatic tumor spheroids (TS) cultured for 13 days in concave microwell 600. Representative images of H&E stained paraffin sections or toluidine blue stained semi-thin sections, SEM and TEM images of Panc-1 (A), Aspc-1 (B) and Capan-2 (C) spheroids. Cross: lipid droplets; N: necrotic regions; I: invagination structure. The scale bars indicate 100 μm, 50 μm, 2 μm and 500 μm, in H&E or toluidine blue stained, SEM, and TEM images, respectively. (TIF) [file pone.0073345.s001.tif]
